# Advancing Programme Science approaches to understand gaps in HIV prevention programme coverage for key populations in 12 Nigerian states: findings from the 2020 Integrated Biological and Behavioural Surveillance Survey

**DOI:** 10.1002/jia2.26269

**Published:** 2024-07-10

**Authors:** Leigh M. McClarty, Kalada Green, Stella Leung, Chukwuebuka Ejeckam, Adediran Adesina, Souradet Y. Shaw, Bronwyn Neufeld, Shajy Isac, Faran Emmanuel, James F. Blanchard, Gambo Aliyu

**Affiliations:** ^1^ Institute for Global Public Health University of Manitoba Winnipeg Manitoba Canada; ^2^ West African Centre for Public Health and Development Abuja Nigeria; ^3^ National Sexually Transmitted and Blood Borne Infection Laboratory Public Health Agency of Canada Winnipeg Manitoba Canada; ^4^ India Health Action Trust Delhi India; ^5^ National Agency for the Control of AIDS Abuja Nigeria

**Keywords:** Nigeria, HIV, Integrated Biological and Behavioural Surveillance Survey, female sex workers, men who have sex with men, people who inject drugs

## Abstract

**Introduction:**

Effective HIV prevention programme coverage is necessary to achieve Nigeria's goal of ending the epidemic by 2030. Recent evidence highlights gaps in service coverage and utilization across the country. The Effective Programme Coverage framework is a Programme Science tool to optimize a programme's population‐level impact by examining gaps in programme coverage using data generated through programme‐embedded research and learning. We apply the framework using Integrated Biological and Behavioural Surveillance Survey (IBBSS) data from Nigeria to examine coverage of four prevention interventions—condoms, HIV testing, and needle and syringe programmes (NSP)—among four key population groups—female sex workers (FSW), men who have sex with men (MSM), people who inject drugs (PWID) and transgender people.

**Methods:**

Data from Nigeria's 2020 IBBSS, implemented in 12 states, were analysed to examine HIV prevention programme coverage among key populations. For each key population group and prevention intervention of interest, weighted IBBSS data were used to retrospectively generate coverage cascades that identify and quantify coverage gaps. Required coverage targets were informed by targets articulated in Nigeria's National HIV/AIDS Strategic Framework or, in their absence, by guidelines from policy normative bodies. Availability‐, outreach‐ and utilization coverage proxy indicators were defined using variables from IBBSS data collection tools. Sankey diagrams are presented to visualize pathways followed by participants between coverage cascade steps.

**Results:**

Required coverage targets were missed for HIV testing and NSP among all key population groups. Condom availability coverage surpassed required coverage targets among FSW and MSM, while utilization coverage only among FSW exceeded the 90% required coverage target. Outreach coverage was low for all key population groups, falling below all required coverage targets.

**Conclusions:**

Our findings identify critical gaps in HIV prevention programme coverage for key populations in Nigeria and demonstrate non‐linear movement across coverage cascades, signalling the need for innovative solutions to optimize coverage of prevention services. Programme‐embedded research is required to better understand how key population groups in Nigeria access and use different HIV prevention services so that programmes, policies and resource allocation decisions can be optimized to achieve effective programme coverage and population‐level impact.

## 1 INTRODUCTION

Nigeria has among the largest population of people living with HIV globally, comprising nearly one‐fifth of all new acquisitions in the West and central African region [1]. In 2019, an estimated 1.9 million people were living with HIV in the country [2], and adult prevalence estimates range from 1.3% [3] to 2.1% [4]. Although Nigeria's overall HIV incidence has declined over the past two decades, heterogeneity exists across populations and geographies [1, 5]. Globally, key populations—female sex workers (FSW), men who have sex with men (MSM), people who inject drugs (PWID) and transgender people—experience a disproportionate burden of the HIV epidemic [6] due to a multitude of intersecting marginalizing factors [7, 8, 9]. In Nigeria, members of key populations are criminalized [1], which, along with pervasive stigmatization and discrimination [10], shapes and perpetuates oppressive contexts in which the delivery and use of HIV prevention and care become increasingly challenging. Despite comprising just 2−3% of Nigeria's adult population, an estimated 11−30% of new HIV acquisitions occur among members of key populations and their sexual partners [4, 11, 12]. Notably, Nigeria's most recent Integrated Biological and Behavioural Surveillance Survey (IBBSS) estimated HIV prevalence among FSW, MSM, PWID and transgender participants at 15.5%, 25.0%, 10.9% and 28.8%, respectively [13].

Nigeria's Revised National HIV/AIDS Strategic Framework (NSF) 2019−21 set a goal to achieve 90% population coverage—including key populations—of combination prevention interventions by 2020, and 95% coverage by 2030 [14]. Ensuring effective HIV prevention programme coverage is crucial to achieve Nigeria's goal of ending the epidemic by 2030 [14, 15]. However, recent evidence highlights gaps in prevention programme coverage and service utilization across the country. In 2020, Durosinmi‐Etti et al. reported that just 50% of key population members in three prioritized Nigerian states were aware of pre‐exposure prophylaxis (PrEP) and HIV self‐testing, with notable heterogeneity by key population group and geography [16]. This gap suggests that different approaches might be required to equitably prioritize populations and geographies, and ensure appropriate scale and coverage, in order to optimize the population‐level impact of HIV prevention programmes [17, 18, 19, 20].

Nigeria's National HIV/AIDS Strategic Plan 2010−2015 [21] introduced a Minimum Prevention Package Intervention (MPPI) promoting the targeted scale‐up of a standardized combination prevention approach for prioritized groups across the country. While the MPPI sets basic guidelines, variation exists in programme implementation, resulting in inconsistent service coverage across geographies and population groups. This variation is due, in part, to a patchwork of multilateral donors, operating at national‐, state‐ and sub‐state levels to support programming implemented by an assortment of local and international non‐governmental organizations.

In several Nigerian states, “One‐Stop Shops” (OSS) are important access points for HIV prevention services among members of key populations [22, 23], particularly in urban and semiurban areas. In rural areas where prevention programming exists, services are primarily delivered through community‐based organizations. OSS are funded by a multitude of international donors, and most are run by local non‐governmental organizations. They comprise walk‐in prevention and treatment services alongside community‐based outreach platforms linked to drop‐in centres. Prevention services offered through OSS outreach include routine, community‐based HIV rapid testing; free condoms; referrals to sexually transmitted infection testing; and prevention counselling. OSS outreach services are typically offered on a regular, pre‐determined schedule that aims to connect with members of key populations quarterly.

During IBBSS implementation, HIV testing and condoms were widely available across all 12 states. As of 2020, PrEP was offered through OSS, but was recommended as a prevention strategy only for “male sex workers and their clients” (14, p. 17). In 2019−20, a pilot needle and syringe programme (NSP) was implemented in three of the 12 IBBSS states—Abia, Gombe and Oyo [24]. By the end of 2022, needle and syringe distribution services were scaled up through NSP in four additional states: Lagos, Rivers, Cross River and Akwa Ibom [25].

The Effective Programme Coverage framework [26] is a Programme Science [27, 28, 29] tool to maximize a public health programme's population‐level impact through iterative cycles of programme‐embedded research, learning and monitoring. Within the framework, four dimensions of programme coverage—required‐, availability‐, contact‐ and utilization coverage (Table S1)—are organized as a “cascade” [30, 31, 32, 33, 34], to routinely identify and measure coverage gaps for programmes’ component interventions [26]. The Effective Programme Coverage framework can guide equity‐focused coverage gap analyses using routine programme monitoring data and simple primary data collection [26], or secondary data sources to generate research agendas aimed at minimizing those gaps. Here, we apply the Effective Programme Coverage framework using information from Nigeria's 2020 IBBSS to retrospectively construct coverage cascades that identify and begin to examine gaps in coverage of three HIV prevention interventions (condoms, HIV testing and NSP) implemented among four key population groups.

## 2 METHODS

### 2.1 Study setting and context

In 2020, following global biobehavioural survey guidelines [35], Nigeria undertook its fourth IBBSS to assess the current state of its HIV epidemic and response among four key population groups: FSW, MSM, PWID, and for the first time, transgender people [13]. The IBBSS was implemented in 12 states across Nigeria's six geopolitical zones (Figure S1)—Kaduna and Kano (North‐West), Benue and Nasarawa (North‐Central), Taraba and Gombe (North‐East), Lagos and Oyo (South‐West), Akwa Ibom and Rivers (South‐South), and Abia and Anambra (South‐East). Sampled states were prioritized based on the most recent HIV prevalence estimates [3], availability of mapping data and size estimates for key population groups [36], and trends observed during previous national surveys [13]. Sampling and data collection methods are described in Additional File 1, and implementation procedures are detailed elsewhere [13]. Briefly, in each IBBSS state, a multistage, probability‐based sampling approach was used to randomly select a representative sample of each key population group from validated “locations” (i.e. spaces where individuals within high‐risk sexual and injecting networks meet their sexual and/or injecting partners/clients and/or engage in activities that increase the likelihood for HIV acquisition). A list of locations had been generated through a programmatic mapping [37, 38] exercise conducted in 2018 [39] and was re‐validated prior to IBBSS sample selection. Because mapping had not been previously carried out for transgender communities in Nigeria, a rapid mapping exercise was conducted before sampling.

### 2.2 Data sources

Biobehavioural surveys are commonly used to inform programmatic decisions and planning [35]. IBBSS data collected between October and December 2020 were used to retrospectively construct programme coverage cascades for three component interventions implemented for key populations as a part of Nigeria's Revised NSF 2019−20 [14].

### 2.3 Required coverage targets

Where possible, required coverage targets for each intervention and key population group were aligned with Nigeria's Revised NSF [14] or the earlier 2017−21 NSF [12]. If national guidance was not available, required coverage targets were based on guidelines outlined by the World Health Organization (WHO) and UNAIDS [40, 41, 42].

### 2.4 Condoms

Required coverage for condoms was set to 90% for FSW, MSM, PWID and transgender participants, as defined in the Revised NSF 2019−21 [14].

### 2.5 HIV testing

Nigeria's NSF 2017−21 set an HIV testing target of 100% for all key populations [12]. The WHO recommends that all members of key populations not previously diagnosed with HIV should have at least one HIV test in a 12‐month period [40]. For each key population group, required coverage targets for HIV testing were set to 100% of the sample reporting no previous HIV test or no knowledge of a previous positive test result.

### 2.6 Needle and syringe programme

Coverage of NSP was assessed for PWID only. While the Revised NSF names NSP as a priority prevention strategy for PWID, it does not provide a specific target for NSP coverage. As such, the required coverage target was aligned with the HIV Prevention Road Map [41], which states that 90% of all PWID should have access to and use NSP services.

### 2.7 Availability‐, outreach‐ and utilization coverage indicators

IBBSS questions were used to generate indicator estimates for the availability‐, contact‐ and utilization coverage steps of the coverage cascade for each key population group. An iterative consultation process with researchers and stakeholders involved in IBBSS tool development and data collection informed the selection of the most appropriate proxy measures. Due to inherent limitations of the IBBSS data collection tool, the contact coverage element of the Effective Programme Coverage framework is re‐framed as *outreach coverage* to more accurately reflect the data available to generate indicator estimates. Indicator definitions for each coverage cascade step, by intervention and key population group, are presented in Table 1.

**Table 1:** Definitions for HIV prevention programme coverage cascade indicator, by component intervention and key population group

		Condoms	HIV testing	Needle and syringe programme
**Availability coverage**	*Female sex workers* *Men who have sex with men* *People who inject drugs*	Knows of a place or person from which they can obtain male condoms.	Knows of a health facility or place in their community where one can receive counselling and testing for HIV.	– – Able to obtain new, unused needles and syringes every time they need them.
	*Transgender people*			–
**Outreach coverage**	*Female sex workers* *Men who have sex with men* *People who inject drugs*	Contacted by a peer educator or by an outreach worker to provide HIV/AIDS‐related services in the past 12 months.		– – Contacted by a peer educator or by an outreach worker to provide HIV/AIDS‐related services in the past 12 months.
	*Transgender people*			–
**Utilization coverage**	*Female sex workers*	Used a condom the last time they had sex with a casual sex partner or a client.	Received most recent HIV test within the past 12 months.	–
	*Men who have sex with men* *People who inject drugs*	Used a condom the last time they had (vaginal or anal) sex with a casual sex partner or a client.		– In the past 1 month, always injected with a needle that no one else had used.
	*Transgender people*			−

### 2.8 Data analyses

Unless otherwise specified, standardized weights were applied to all analyses to account for the survey's multistage, stratified sampling approach outlined in Additional File 1. Coverage cascades were retrospectively constructed for condoms and HIV testing among FSW, MSM, PWID and transgender participants, and for NSP among PWID, using weighted proportions. For condom and NSP analyses, all coverage cascade indicators were calculated as a weighted proportion of the total sample size of relevant key population groups. For HIV testing, denominators for coverage indicators included all participants who reported no previous HIV test or no known previous positive HIV test result. Each coverage cascade indicator was calculated independently of the previous coverage dimension, such that individuals were not required to be included in a previous dimension to be counted in the subsequent. All coverage cascade analyses were performed in SAS 9.4.

For each prevention intervention and relevant key population group, Sankey diagrams were constructed using weighted IBBS data to visualize participant pathways between coverage cascade dimensions, beginning at availability‐ and ending at utilization coverage. Coverage cascade dimensions within Sankey diagrams are “nodes,” while bands depicting the path between two nodes are “links.” The size of node elements represents the relative proportion of participants within the coverage dimensions and the width of the link elements represents the relative proportions of individuals following pathways between nodes. Sankey diagrams were generated with R Statistical Software (v4.3.1; R Core Team 2023) using the ggplot2 (v3.4.4; Wickham H, 2016), ggsankey (v0.0.99999; Sjoberg D, 2023) and dplyr (v1.1.3; Wickham H et al., 2023) packages.

### 2.9 Ethical considerations

Informed consent was sought from all IBBSS participants prior to initiating the behavioural and biological components of the study. This work was reviewed and approved by the National Health Research Ethics Committee, Abuja, Nigeria (NHREC/01/01/2007‐25/08/2020) and the Health Research Ethics Board (HS23933) at the University of Manitoba, Canada.

## 3 RESULTS

In total, 17,975 participants—4974 FSW, 4397 MSM, 4414 PWID and 4190 transgender people—took part in the 2020 IBBSS study and were included in coverage cascade and Sankey analyses for condom and NSP interventions. Of those, 4735 FSW, 4059 MSM, 4355 PWID and 3965 transgender participants reported no previous HIV test or no knowledge of a previous positive HIV test result; these subsets comprised denominators for HIV testing coverage cascades and Sankey analyses. Unweighted data for select socio‐demographic characteristics are presented, by key population group, in Table 2.

**Table 2:** Characteristics of female sex workers, men who have sex with men, people who inject drugs and transgender participants in the 2020 Integrated Biobehavioural Surveillance Survey in 12 Nigerian states (unweighted data)

	Female sex workers, *n* (%)	Men who have sex with men, *n* (%)	People who inject drugs, *n* (%)	Transgender, *n* (%)
	*n* = 4974	*n* = 4397	*n* = 4414	*n* = 4190
**Age groups (years)**				
15−19	216 (4.3)	559 (12.7)	153 (3.5)	579 (13.8)
20−24	1288 (25.9)	1819 (41.4)	914 (20.7)	1955 (46.7)
25−29	1540 (31.0)	1275 (29.0)	1239 (28.1)	1126 (26.9)
30−34	1017 (20.5)	514 (11.7)	949 (21.5)	346 (8.3)
35−49	865 (17.3)	216 (4.9)	1020 (23.1)	175 (4.2)
50+	48 (1.0)	14 (0.3)	139 (3.2)	9 (0.2)
**Sex**				
Female	−	−	520 (11.8)	−
Male			3889 (88.1)	
Transgender			5 (0.1)	
**Sex assigned at birth**				
Female	−	−	−	537 (12.8)
Male				3653 (87.2)
**Highest level of education**				
Completed tertiary	501 (10.1)	1447 (32.9)	988 (22.4)	1271 (30.3)
Completed secondary	2181 (43.9)	2298 (52.3)	2005 (45.4)	2262 (54.0)
Completed primary	524 (10.5)	83 (1.9)	347 (7.9)	102 (2.4)
Some secondary	1103 (22.2)	470 (10.7)	680 (15.4)	478 (11.4)
Some primary	243 (4.9)	41 (0.9)	158 (3.6)	20 (0.5)
Quranic education only	90 (1.8)	33 (0.8)	51 (1.2)	31 (0.7)
Never attended school	332 (6.7)	25 (0.6)	185 (4.2)	26 (0.6)
**Employment status**				
Employed full‐time	967 (19.4)	1166 (26.5)	1041 (23.6)	742 (17.7)
Employed part‐time	773 (15.5)	606 (13.8)	1000 (22.7)	852 (20.3)
Full‐time student	168 (3.4)	933 (21.2)	363 (8.2)	891 (21.3)
Unemployed	2830 (56.9)	1284 (29.2)	1488 (33.7)	1181 (28.2)
Retired	5 (0.1)	4 (0.1)	18 (0.4)	85 (2.0)
Other	204 (4.0)	334 (7.6)	408 (9.2)	414 (9.9)
Don't know/No response	32 (0.6)	70 (1.6)	96 (2.2)	25 (0.6)
**Reported clients or exchanging sex for money/gifts**				
Yes	4974 (100)	1219 (27.7)	758 (17.2)	575 (37.6)
**Location typology** ^a^				
Bar/nightclub/casino	1385 (27.8)	758 (17.2)	458 (10.4)	1065 (25.4)
Brothel	1389 (27.9)	−	−	−
Home	404 (8.1)	1028 (23.4)	−	992 (23.7)
Hotel/lodge	1061 (21.3)	802 (18.2)	328 (7.4)	691 (16.5)
Street/public place	579 (11.6)	882 (20.1)	1567 (35.5)	1029 (24.6)
Virtual	−	582 (13.2)	−	−
Bunks	−	−	1408 (31.9)	−
Cemetery/abandoned buildings	−	−	279 (6.3)	−
Other^b^	156 (3.1)	344 (7.8)	374 (8.5)	413 (9.86)

^a^ Missing data for one (0.02%) men who have sex with men participant.

^b^ Other category includes: Female sex workers (FSW)—hostel/campus, others, trailer parks/transport stops; Men who have sex with men (MSM)—eatery/shopping mall, hostel/campus, others; People who inject drugs (PWID)—brothel, home, others, trailer parks/transport stops; Transgender participants—brothel, eatery/shopping mall, hostel/campus, non‐governmental organization (NGO).

**Figure 1:**
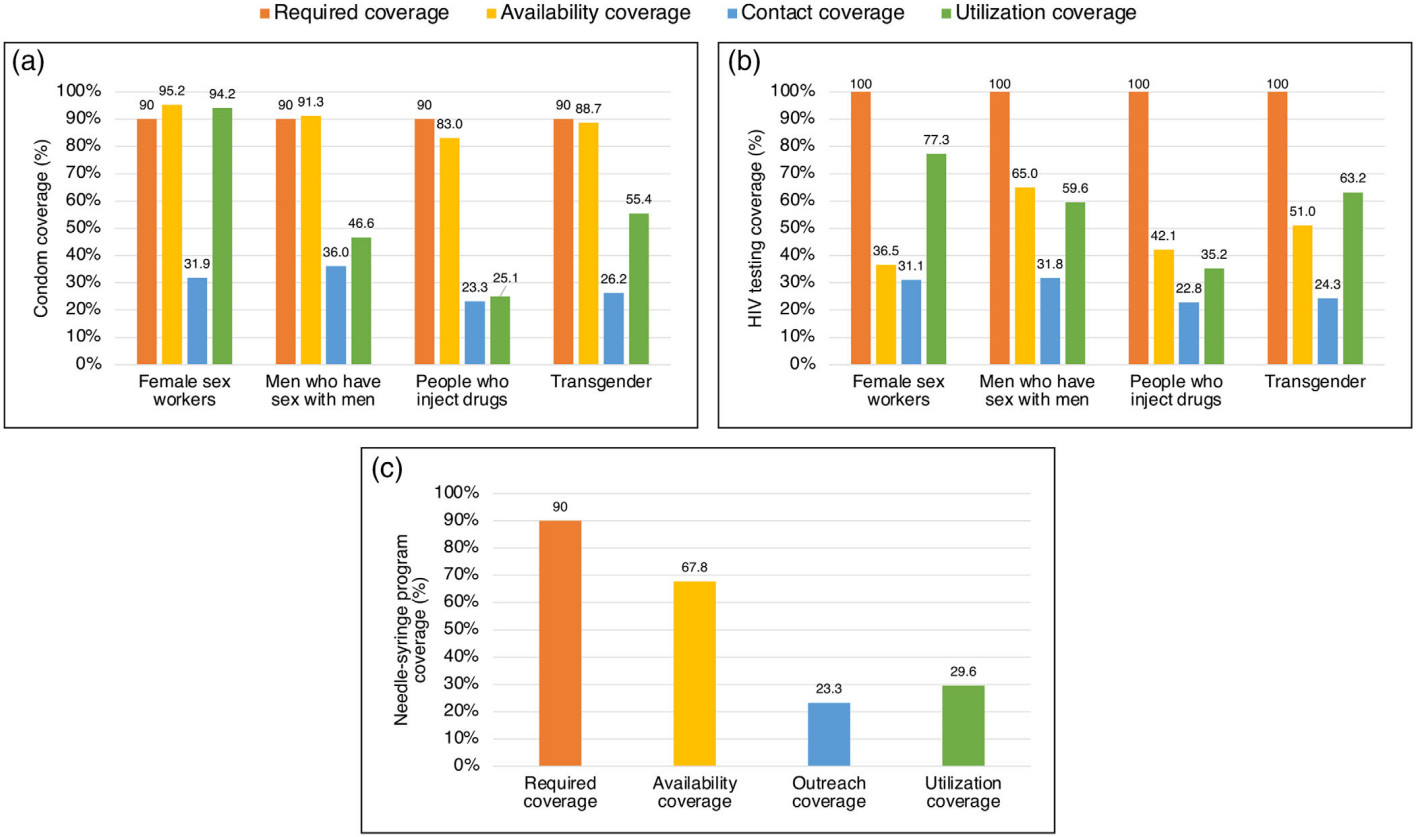
**Coverage cascades for (a) condoms, (b) HIV testing, (c) pre‐exposure prophylaxis among female sex workers, men who have sex with men, people who inject drugs and transgender participants across 12 Nigerian states**.

**Figure 2:**
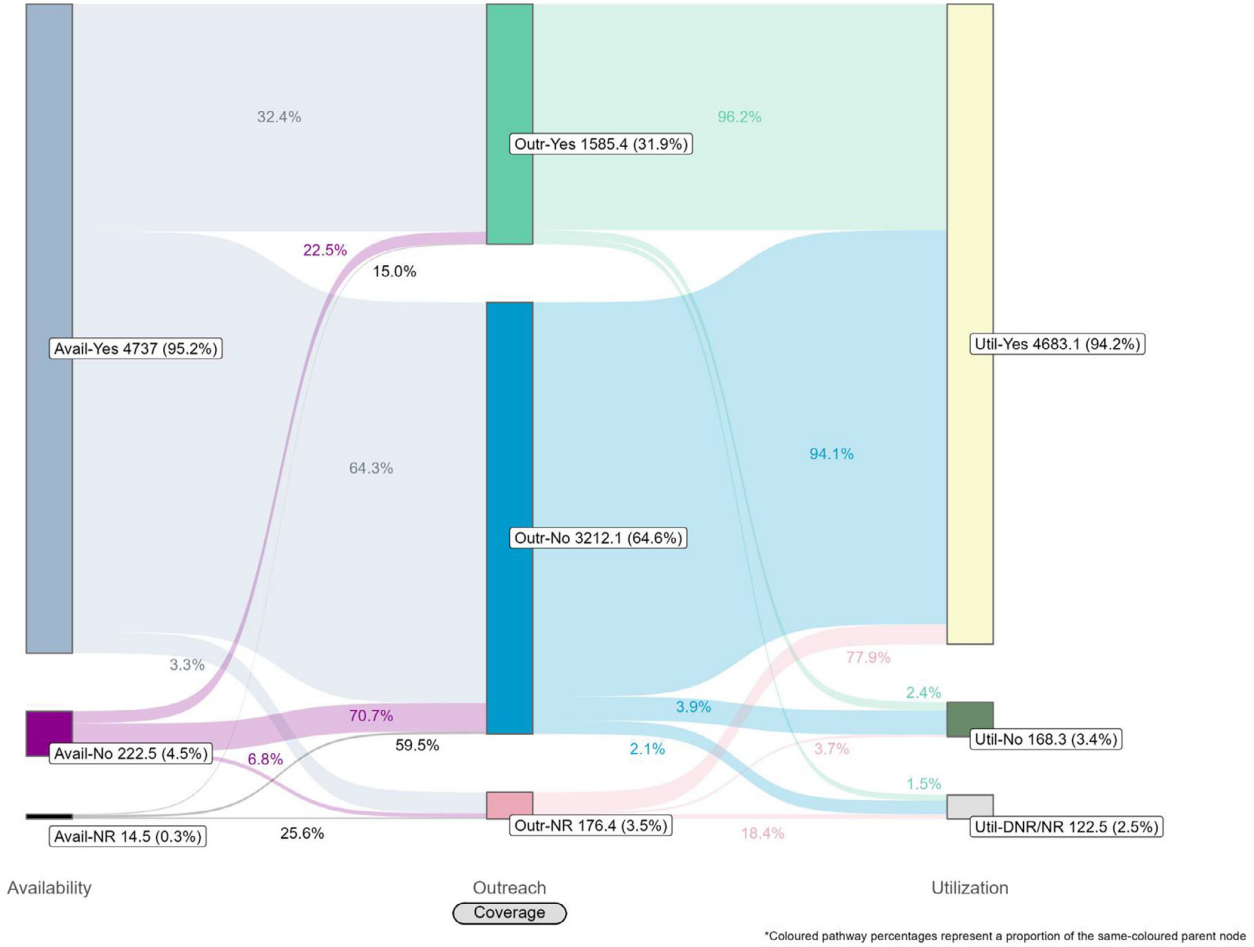
**Pathways of female sex workers through the condom coverage cascade in 12 Nigerian states. Unweighted *N* = 4974**. Abbreviations: DNR/NR, do not remember/no response; NR, no response.

**Figure 3:**
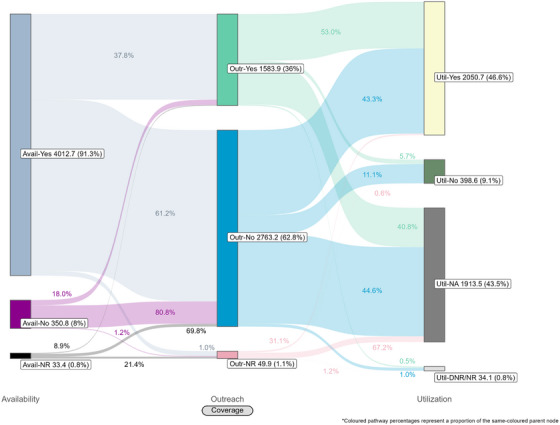
**Pathways of men who have sex with men through the condom coverage cascade in 12 Nigerian states. Unweighted *N* = 4397**. Abbreviations: DNR/NR, do not remember/no response; NR, no response.

**Figure 4:**
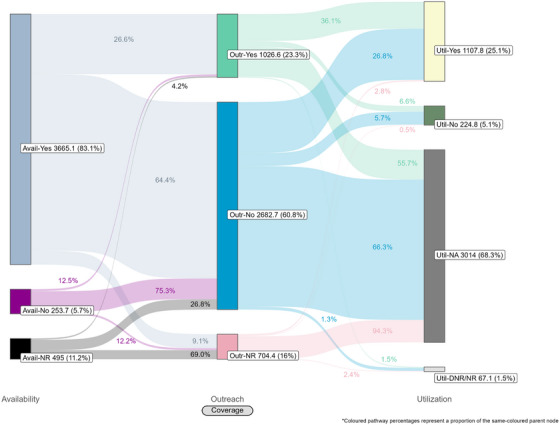
**Pathways of people who inject drugs through the condom coverage cascade in 12 Nigerian states. Unweighted *N* = 4414**. Abbreviations: DNR/NR, do not remember/no response; NA, not applicable; NR, no response.

**Figure 5:**
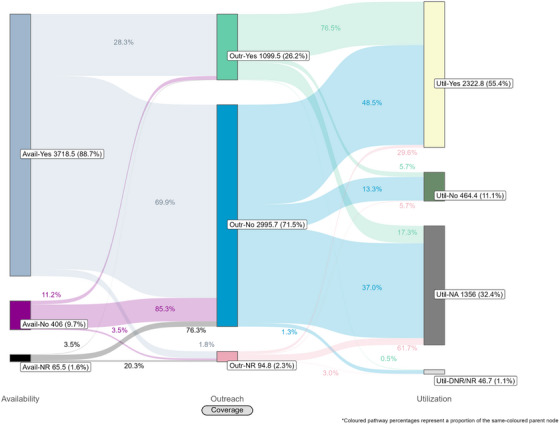
**Pathways of transgender participants through the condom coverage cascade in 12 Nigerian states. Unweighted *N* = 4190**. Abbreviations: DNR/NR, do not remember/no response; NA, not applicable; NR, no response.

**Figure 6:**
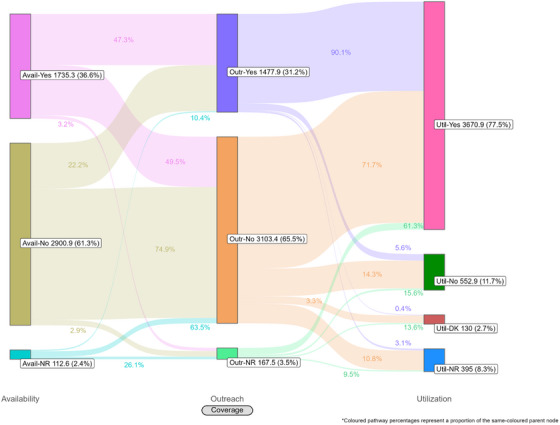
**Pathways of female sex workers through the HIV testing coverage cascade in 12 Nigerian states. Unweighted *N* = 47,35**. Abbreviations: DK, do not know; NR, no response.

**Figure 7:**
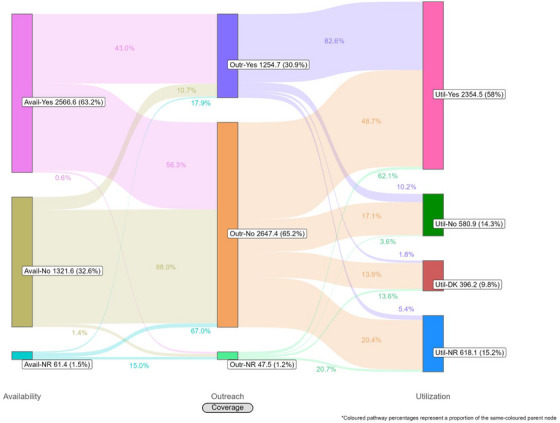
**Pathways of men who have sex with men through the HIV testing coverage cascade in 12 Nigerian states. Unweighted *N* = 4059**. Abbreviations: DK, do not know; NR, no response.

**Figure 8:**
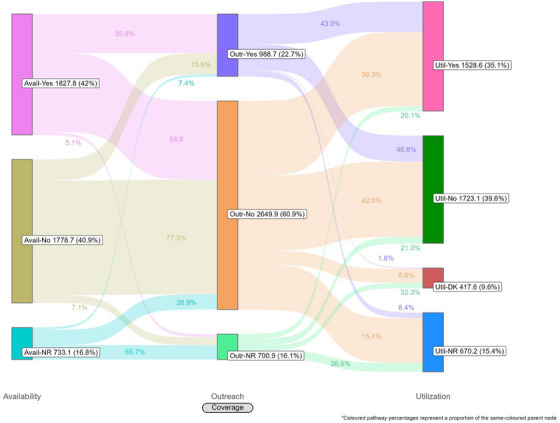
**Pathways of people who inject drugs through the HIV testing coverage cascade in 12 Nigerian states. Unweighted *N* = 4355**. Abbreviations: DK, do not know; NR, no response.

**Figure 9:**
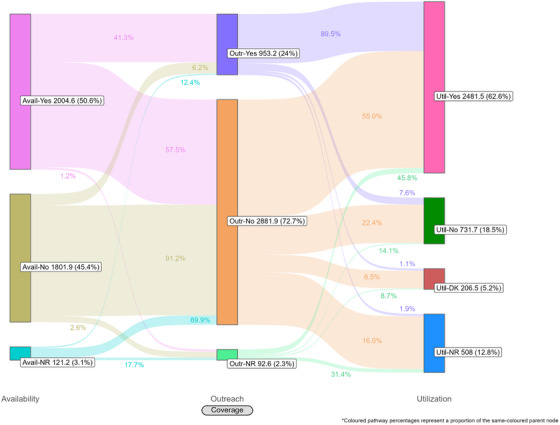
**Pathways of transgender participants through the HIV testing coverage cascade in 12 Nigerian states. Unweighted *N* = 3965**. Abbreviations: DK, do not know; NR, no response.

**Figure 10:**
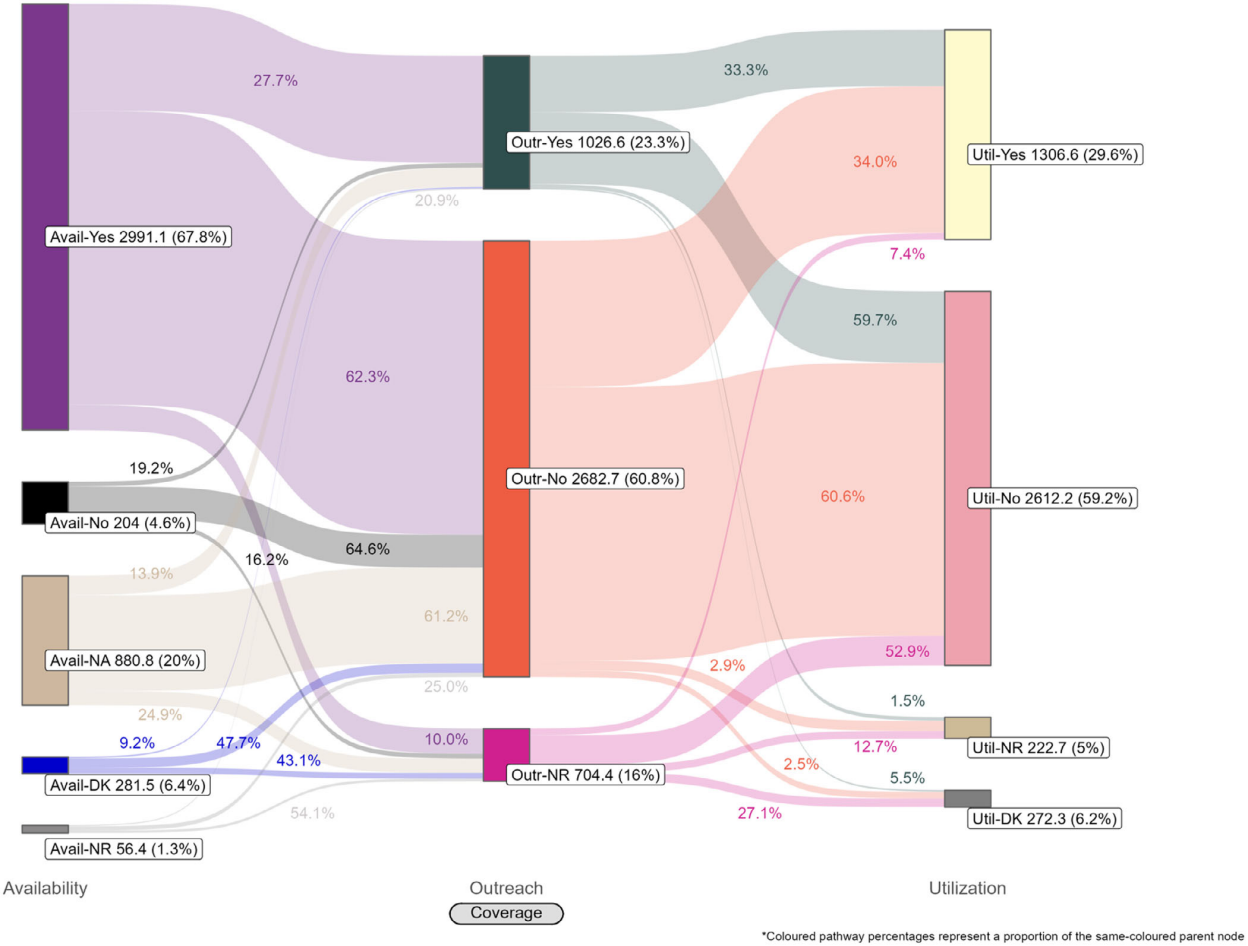
**Pathways of people who inject drugs through the needle and syringe programme coverage cascade in 12 Nigerian states. Unweighted *N* = 4414**. Abbreviations: DK, do not know; NA, not applicable; NR, no response.

Coverage cascades for condoms and HIV testing among all key population groups are presented in Figure 1 (panels A and B, respectively) and for NSP among PWID in panel C. Presented Sankey diagrams depict the pathways of FSW, MSM, PWID and transgender participants through the condom (Figures 2, 3, 4, 5) and HIV testing (Figures 6, 7, 8, 9) coverage cascades, and the NSP coverage cascade among PWID participants (Figure 10).

### 3.1 Condom coverage

Reported availability coverage for condoms exceeded the 90% required coverage target for FSW (95.2%) and MSM (91.3%), while outreach coverage was consistently below target (31.9% and 36.0%, respectively; Figure 1, panel A). Among FSW, utilization coverage (94.2%) surpassed the required coverage target but fell below 60% for all other groups. Condom utilization coverage among PWID was notably low at 25.1%.

Sankey diagrams highlight pathways from condom availability coverage through utilization coverage for FSW (Figure 2), MSM (Figure 3), PWID (Figure 4) and transgender participants (Figure 5). Relatively few participants reported outreach coverage without reporting condom availability coverage (FSW: 22.5%, MSM: 18.0%, PWID: 12.5% and transgender: 11.2%). Among FSW reporting no outreach coverage, 94.1% reported utilization coverage, which comprised over half of all FSW reporting condom utilization coverage. Approximately one‐quarter (26.8%) of PWID reported utilization coverage despite no contact with outreach services in the past year, whereas nearly half of MSM and transgender participants reported the same (43.3% and 48.5%, respectively).

### 3.2 HIV testing coverage

For all key population groups, HIV testing coverage did not reach the required coverage target (100%) for those recommended for annual testing (Figure 1, panel B). Most FSW (77.3%), MSM (59.6%) and transgender (63.2%) participants reported an HIV test in the past 12 months, while 35.2% of PWID participants reported the same. Among FSW and transgender participants, utilization coverage (77.3% and 63.2%, respectively) exceeded availability coverage (36.5% and 51.0%, respectively).

HIV testing Sankey diagrams illustrate that regardless of reported availability coverage, over half of participants from all groups reported no outreach coverage (FSW: 65.5%, MSM: 65.2%, PWID: 60.9% and transgender: 72.7%). Most FSW (Figure 6), MSM (Figure 7) and transgender participants (Figure 9) reporting outreach coverage also reported utilization coverage (90.1%, 82.6% and 89.5%, respectively), whereas just 43.0% of PWID reporting outreach coverage had received their last HIV test in the past 12 months (Figure 8). Most FSW (71.7%) and transgender participants (55.0%) who did not report outreach coverage still reported utilization coverage. Regardless of overall utilization coverage among each key population group, the majority reporting an HIV test in the past 12 months did not report outreach coverage in the same period, represented by the thicker links connecting the two nodes.

### 3.3 NSP coverage

NSP required coverage targets (90%) were not met for availability (67.8%), outreach (23.3%), nor utilization coverage (29.6%) among PWID participants (Figure 1, panel C).

The NSP Sankey diagram (Figure 10) indicates that a majority of PWID participants reported availability coverage (67.8%) for NSP and no outreach coverage (60.8%), while less than one‐third (29.6%) used a new needle and syringe every time they injected in the past month. Approximately equal proportions of those who reported (34.0%) and did not report (33.3%) outreach coverage also reported utilization coverage.

## 4 DISCUSSION

Coverage cascade analyses of Nigeria's 2020 IBBSS data identified several missed coverage targets. Required coverage targets were not met for condoms among MSM, PWID and transgender participants; HIV testing among FSW, MSM, PWID and transgender participants; nor NSP among PWID. These findings align with previous studies from Nigeria suggesting relatively low uptake of HIV prevention services among key population groups [16, 25, 43, 44]. Our findings also indicate low outreach coverage for all key population groups, falling well below the required coverage targets for each intervention. In Nigeria, outreach‐based platforms are commonly configured as planned periods of contact, such that individual outreach workers only aim to meet each of their constituents once per quarter, over three to four scheduled outreach activities. The 2020 IBBSS was conducted in the midst of the COVID‐19 pandemic [13], prior to the rollout of vaccinations [45], during which time federal public health recommendations for physical distancing and limited in‐person contact were actively enforced [46]. Thus, it is plausible that the quarterly outreach model, in combination with pandemic‐related restrictions, might partially explain low reported outreach coverage. Despite this, utilization of condom and HIV testing services—both primarily delivered through outreach in Nigeria—exceeded outreach coverage among all key population groups (Figure 1, panels A and B). This could suggest that, during the IBBSS data collection period coinciding with pandemic‐related restrictions, participants accessed certain prevention services through platforms not mediated by outreach.

These analyses provide an important opportunity to understand why identified coverage gaps exist and conceptualize strategies for addressing them in hopes of improving all dimensions of programme coverage. A noted limitation of cascade models is the implication that “ideal” or typical progress along the continuum should be a sequential and linear pathway [47, 48]. Accordingly, the coverage cascade within the Effective Programme Coverage framework [26] cannot be accurately conceptualized as a linear pathway towards effective programme coverage. Sankey diagrams highlight that a larger proportion of IBBSS participants reporting condom use at last sex with higher‐risk sexual partners (Figures 2, 3, 4, 5) or annual HIV testing (Figures 6, 7, 8, 9) also reported that they had not had contact with a peer or outreach worker in the last 12 months. These findings emphasize a need to closely examine and better understand the role of outreach‐based contact as a precursor to effective uptake of prevention services across contexts and key population groups. Although condoms are freely available per the MPPI, previous studies suggest a willingness to pay for condoms among men in Nigeria [49, 50], and an increased likelihood of consistent condom use when HIV knowledge is relatively strong [51]. It is plausible that members of key population groups, who generally receive intensified HIV prevention messaging, could be acquiring condoms independent of outreach‐based services. Similarly, HIV testing is widely promoted among key populations and while primarily accessed through outreach‐based platforms, they are also readily available via non‐outreach‐based modalities, including government and private facilities, pharmacies, mobile clinics and self‐testing options across Nigeria [15, 52, 53, 54, 55, 56].

### 4.1 Implications for programming and future analyses

Community‐ and peer‐led outreach is a well‐established, integral component of comprehensive HIV prevention programmes for key and priority populations [57, 58]. It enhances availability, accessibility and uptake of prevention services, and critically contributes to community empowerment [59] and economic mobility [60]. However, our findings highlight potential variability in the role of outreach in optimizing programme coverage, which warrants further investigation. Following a Programme Science approach, the Effective Programme Coverage framework underscores the need for programmes to calibrate the intensity of contact with different service users to align with needs, the maturity of the prevention programme and the newness of the interventions [26]. Microplanning is one effective strategy to optimize outreach efforts in evolving contexts for improved programme coverage [61, 62, 63]. For example, more frequent or different modes of outreach might be necessary to increase uptake of newer, more complex prevention interventions such as PrEP and NSP, or to engage with key populations facing increased stigmatization and criminalization. Whereas long‐standing, widely available and generally accepted prevention strategies, such as condoms and routine HIV testing, might require relatively less intense contact outreach coverage to achieve desired levels of utilization coverage.

Although PrEP coverage analyses were beyond the scope of this work, preliminary analyses of 2020 IBBSS data indicated low availability and utilization coverage among all key population groups, regardless of outreach coverage. PrEP is a relatively new and complex intervention, requiring daily dosing and management of potential side effects. As such, interactions with peer educators and outreach workers might be an important facilitator of PrEP uptake. Indeed, evidence from studies among key populations in Nigeria and India demonstrate that engagement with peer education and linkages to community networks facilitate PrEP acquisition and use [16, 64, 65]. Understanding the contexts in which refinements to prevention programmes’ outreach strategies will yield greater impact and optimize effective coverage should be prioritized.

Our findings provide a platform upon which to develop a programme‐embedded research agenda to inform refinements to Nigeria's HIV prevention strategy. Coverage gap analyses are strengthened when examined through an equity lens, which can illuminate intersections of vulnerabilities within and between key population groups and provide invaluable information to programmes for adjusting strategies to optimize service delivery. Future work will stratify coverage gap analyses by age, geography, duration identifying with a key population group and location typology. Moving forward, additional qualitative and quantitative studies will be necessary to clarify the mechanisms and pathways through which key population groups access, contact and use prevention programmes to inform improvements in policy and programme design and optimize resource allocation.

### 4.2 Limitations

As with any survey, IBBSS data might be overestimating programme coverage, including prevention intervention utilization, due to social desirability and confirmation biases. Coverage cascades and Sankey diagrams were generated through post hoc analyses with data from a survey that was not explicitly designed to measure HIV prevention programme coverage, thus limiting the selection of appropriate variables and impacting the certainty of our interpretations. The 2020 IBBSS lacked an appropriate proxy variable for facility‐ and other non‐outreach‐based contact with the national prevention programme, which could have been a more sensitive contact coverage indicator.

Additionally, our assessment of NSP coverage might not be representative of all 12 IBBSS states as harm reduction services had not been uniformly scaled up at the time of survey implementation. NSP services have been scaled up across Nigeria since IBBSS implementation, thus our findings could be an underrepresentation of current reality. Embedded research approaches [26] and newer methods, such as expanded Polling Booth Surveys [66, 67, 68], facilitate rapid and less resource‐intensive data collection that can be implemented more frequently than IBBSS to assess programme coverage and outcomes and inform timely adjustments to programming to optimize population‐level impact.

Finally, Nigeria's Revised NSF 2019−21—in place during IBBSS implementation—recommended PrEP as a prevention strategy only for male sex workers and their clients [14]. The 2020 IBBSS did not intentionally sample for self‐identifying male sex workers, so do not present PrEP coverage analyses. However, the most recent 2021−25 National HIV and AIDS Strategic Framework recommends PrEP for all key population groups, with a coverage target of 20% by 2025 [15]. Future population‐based studies examining programme coverage in Nigeria should incorporate variables to appropriately measure PrEP coverage.

## 5 CONCLUSIONS

The Effective Programme Coverage framework can guide the development of embedded research agendas that generate new knowledge from programme‐derived questions. Evidence can be reintegrated back into programmes to refine strategies around intervention mixes and service delivery, with the aim of yielding improved, more equitable programme outcomes and optimizing population‐level impact [26].

Analysing 2020 IBBSS data using the coverage cascade construct highlights critical gaps in HIV prevention programme coverage across 12 Nigerian states and Sankey diagrams clearly underscore non‐linear movement across the cascades. Underlying assumptions of linearity within cascade models can obscure complexities underlying gaps in programme coverage. Importantly, efforts to improve coverage of HIV prevention services will inevitably be impeded in policy and legal contexts, such as those in Nigeria, that continue to criminalize key populations or the environments in which they work [69, 70, 71]. Following a Programme Science approach and building off of the presented findings, further embedded research is required to elucidate how key population groups in Nigeria come to know about and navigate HIV prevention services so that programmes, policies and resource allocation decisions can be optimally implemented to maximize effective programme coverage and achieve population‐level impact.

## COMPETING INTERESTS

SYS holds a Tier 2 Canada Research Chair (CRC) in Programme Science and Global Public Health. JFB holds a Tier 1 CRC in Epidemiology and Global Public Health. LMM, KG, SL, CE, AA, BN, SI, FE and GA declare that they have no competing interests.

## AUTHORS’ CONTRIBUTIONS

LMM conceived of, designed and conducted analyses, and drafted the manuscript. KG, CE, AA, SI, FE and GA contributed to the design and implementation of the study, acquisition of data and critically revised the manuscript. SL conducted analyses. BN supported the first draft of the manuscript. SYS and JFB contributed to the conception and interpretation of data analyses and critically revised the manuscript. All authors approved the final version of the manuscript.

## Supporting information


**Figure S1**: Map of Nigeria highlighting the twelve states across six geopolitical zones included in the 2020 Integrated Biological and Behavioural Surveillance Survey

**Table S1**: Coverage cascade steps definitions, as defined in the Effective Programme Coverage framework

**Additional File 1**: Overview of sampling strategy used in the 2020 Integrated Biological and Behavioural Surveillance Survey in twelve Nigerian States

## Data Availability

Data from the 2020 IBBSS that support the findings of this study are under the stewardship of NACA; any requests to access IBBSS data should be directed to the Office of the Director General NACA. The data are not publicly available due to privacy and ethical restrictions, as they contain sensitive information pertaining to criminalized populations.
