# Supplementary material for: Advancing Programme Science approaches to understand gaps in HIV prevention programme coverage for key populations in 12 Nigerian states: findings from the 2020 Integrated Biological and Behavioural Surveillance Survey
Source: J Int AIDS Soc. 2024 Jul 10;27(Suppl 2):e26269. doi: 10.1002/jia2.26269 (PMC11236907; doi:10.1002/jia2.26269)
Supplement: Supplementary file 1 — Figure S1: Map of Nigeria highlighting the twelve states across six geopolitical zones included in the 2020 Integrated Biological and Behavioural Surveillance Survey [file JIA2-27-e26269-s001.docx]

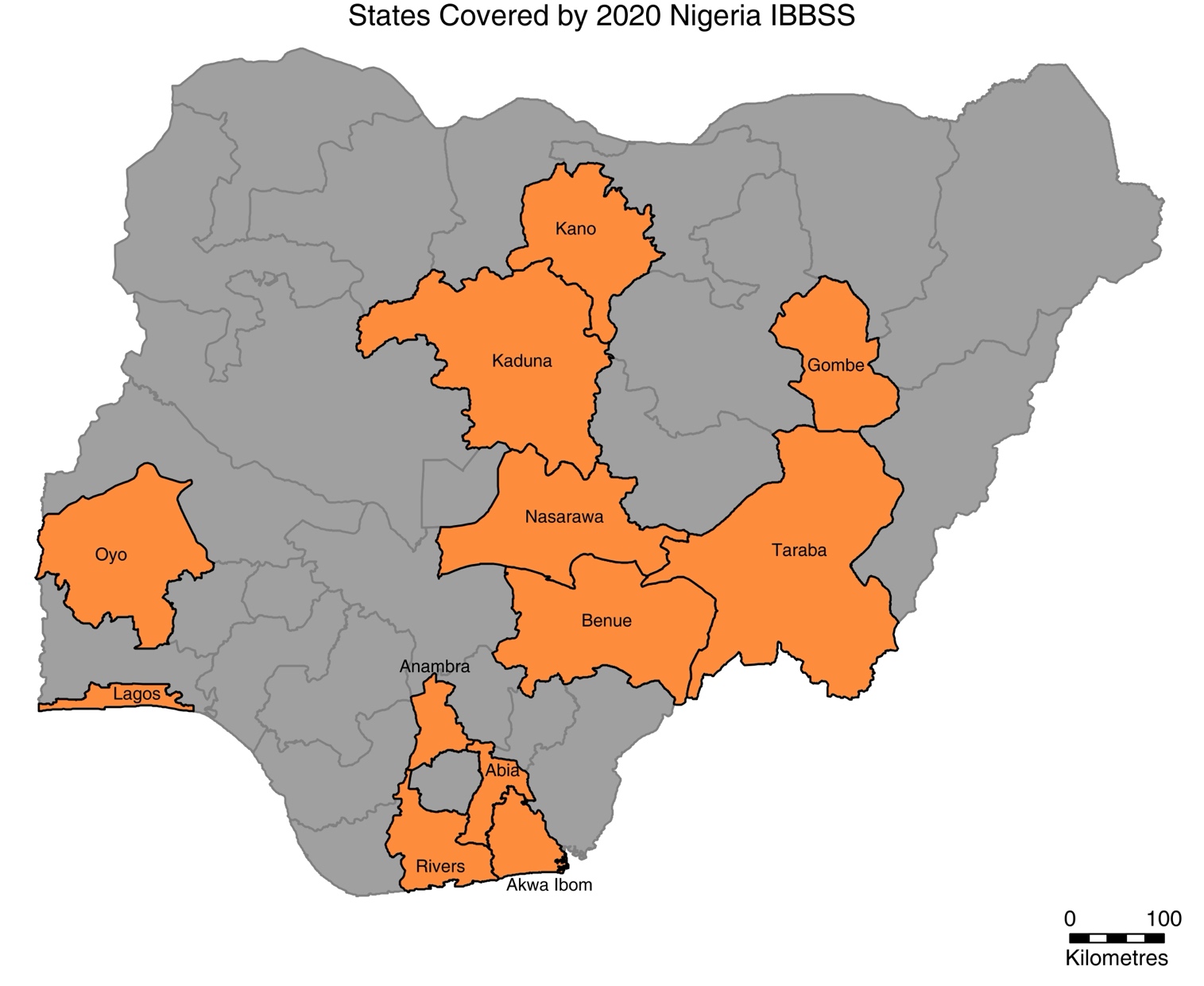


**Figure S1.** Map of Nigeria highlighting the twelve states across six geopolitical zones included in the 2020 Integrated Biological and Behavioural Surveillance Survey.
