# Supplementary material for: Advancing Programme Science approaches to understand gaps in HIV prevention programme coverage for key populations in 12 Nigerian states: findings from the 2020 Integrated Biological and Behavioural Surveillance Survey
Source: J Int AIDS Soc. 2024 Jul 10;27(Suppl 2):e26269. doi: 10.1002/jia2.26269 (PMC11236907; doi:10.1002/jia2.26269)
Supplement: Supplementary file 2 — Table S1: Coverage cascade steps definitions, as defined in the Effective Programme Coverage framework [file JIA2-27-e26269-s003.docx]

# Supplementary table

**Table S1.** Coverage cascade steps definitions, as defined in the Effective Programme Coverage framework (26).

| Coverage cascade step | Definition |
| --- | --- |
| Required coverage | - The first and foundational step within the coverage cascade; acts as the denominator for all subsequent cascade steps. - A programmatic target informed by a public program’s goals and established as a part of a program’s strategy. - A quantifiable metric that defines the populations that benefit most from programme services, within a given context (considering factors such as geography, local epidemiology, and epidemic phase). - Establishes the target for programme outputs and outcomes that are monitored during implementation. |
| Availability coverage | - Within a public health program, represents the program’s capacity to provide services to prioritized populations who require programme coverage. - Measured by the proportion of programme components available to prioritized populations (and subpopulations) within prioritized geographies, to meet required coverage targets. - Estimates for availability coverage can often be based on resources or funding available to a particular programme. - Availability coverage targets for different programme services or interventions should be set separately as need for each will differ by population, geography, and epidemiological context. |
| Contact coverage | - Measures contact between programs and prioritized populations, relative to the required coverage target. - Intensity (e.g., frequency and consistency of services) is a key dimension of contact coverage and should accommodate differing levels of assessed risk or need among prioritized populations. |
| Utilization coverage | - Measures uptake of programme services by prioritized populations in relation to the required coverage target. - Compared to contact coverage, utilization coverage will be more directly related to observed changes in monitored programme outcomes. |
