# Supplementary material for: Advancing Programme Science approaches to understand gaps in HIV prevention programme coverage for key populations in 12 Nigerian states: findings from the 2020 Integrated Biological and Behavioural Surveillance Survey
Source: J Int AIDS Soc. 2024 Jul 10;27(Suppl 2):e26269. doi: 10.1002/jia2.26269 (PMC11236907; doi:10.1002/jia2.26269)
Supplement: Supplementary file 3 — Additional File 1: Overview of sampling strategy used in the 2020 Integrated Biological and Behavioural Surveillance Survey in twelve Nigerian States [file JIA2-27-e26269-s002.docx]

**Additional File 1.** Overview of sampling strategy used in the 2020 Integrated Biological and Behavioural Surveillance Survey in twelve Nigerian States.

# Sampling strategy

A separate sampling frame was developed for each key population group included in the 2020 IBBSS, based upon mapped locations (i.e., spaces where individuals within high-risk sexual and injecting networks meet their sexual and/or injecting partners/clients and/or engage in behaviours that increase likelihood for HIV acquisition) and population size estimates established in 2018 for FSW, MSM, and PWID. Mapping and size estimation exercises for transgender communities had not been previously conducted in Nigeria. Prior to 2020 IBBSS implementation, a rapid mapping exercise was carried out through interviews with members of FSW, MSM, and transgender communities, as well as key informants at community-based organizations providing services for transgender communities in the IBBSS states.

First, a rapid validation process was conducted to ensure that locations identified through the previous mapping exercise (i.e., the ‘universe’ for the sampling frame) were still active. Any newly identified locations were added to the validated list and any inactive locations were removed. Both virtual and physical locations were mapped for MSM, while only physical locations were mapped for FSW, PWID, and transgender participants. Next, a multistage population-based sampling approach was used to generate a random sample of potential participants from each key population group, with the selection of random locations occurring in stage 1 and the random selection of participants from the selected locations in stage 2.

## Stage 1: Location selection

In stage 1, for each key population group surveyed, a fixed number of locations (i.e., the primary sampling units, PSU) were randomly selected from the list of re-validated locations, after stratifying by local government areas (i.e., sub-state level geographical units), location ‘typology’, and location size (i.e., estimated average number of key population members on a location on a typical day). The number of locations per key population group was determined based on the required sample size and the location size. This resulted in a total of 104, 92, and 93 PSU for FSW, PWID and transgender individuals, respectively. As both physical and virtual locations were considered in generating the MSM sample, the per state sample size of 372 was proportionally distributed according to the estimated number of physical and virtual spots in each state (as determined through location validation and virtual mapping exercises). The number of physical and virtual PSU per state was decided in proportion to the estimated population sizes of MSM in each of these typologies. The sample size within each selected PSU was fixed in proportion to the estimated size of each key population at the selected location.

## Stage 2: Selection of participants

Once validated locations (PSU) from which participants were to be sampled had been randomly selected, a pre-determined number of participants were randomly sampled from each PSU. The required sample of participants from each PSU was determined through a probability-proportionate-to-size based on the estimated location size. Trained community members were engaged as social mobilizers to identify eligible individuals from each key population group within each PSU, and a systematic, random sample of the required number of participants were selected from those eligible in each PSU. Randomly selected individuals who were eligible to participate in the study were taken through an informed consent process before initiating the IBBSS. If a potential participant declined to move forward with the study, the next eligible individual was approached. For virtual MSM PSU, random selection and recruitment was conducted online and the IBBSS was conducted at a pre-arranged physical location.
